# Drug resistance in multiple myeloma: latest findings and new concepts on molecular mechanisms

**DOI:** 10.18632/oncotarget.1497

**Published:** 2013-11-24

**Authors:** Jahangir Abdi, Guoan Chen, Hong Chang

**Affiliations:** ^1^ Division of Immunopharmacology, Utrecht Institute for Pharmaceutical Sciences, Utrecht University, Utrecht, The Netherlands; ^2^ Dept. of Hematology & Oncology, The First Affiliated Hospital of Nanchang University, Nanchang, China; ^3^ Division of Molecular and Cellular Biology, Toronto General Research Institute; ^4^ Dept. of Laboratory Medicine & Pathobiology, University of Toronto; ^5^ Dept. of Laboratory Hematology, University Health Network, Toronto, Ontario, Canada

**Keywords:** multiple myeloma, drug resistance, signaling pathways, oncogenes

## Abstract

In the era of new and mostly effective therapeutic protocols, multiple myeloma still tends to be a hard-to-treat hematologic cancer. This hallmark of the disease is in fact a sequel to drug resistant phenotypes persisting initially or emerging in the course of treatment. Furthermore, the heterogeneous nature of multiple myeloma makes treating patients with the same drug challenging because finding a drugable oncogenic process common to all patients is not yet feasible, while our current knowledge of genetic/epigenetic basis of multiple myeloma pathogenesis is outstanding. Nonetheless, bone marrow microenvironment components are well known as playing critical roles in myeloma tumor cell survival and environment-mediated drug resistance happening most possibly in all myeloma patients. Generally speaking, however; real mechanisms underlying drug resistance in multiple myeloma are not completely understood. The present review will discuss the latest findings and concepts in this regard. It reviews the association of important chromosomal translocations, oncogenes (e.g. TP53) mutations and deranged signaling pathways (e.g. NFκB) with drug response in clinical and experimental investigations. It will also highlight how bone marrow microenvironment signals (Wnt, Notch) and myeloma cancer stem cells could contribute to drug resistance in multiple myeloma.

## INTRODUCTION

Multiple myeloma (MM) is the second most common but as yet incurable hematologic malignancy characterized by infiltration in the bone marrow of malignant plasma cells. MM is usually preceded by a pre-malignant stage termed monoclonal gammopathy of undetermined clinical significance (MGUS) which progresses to overt MM at a rate of 0.5% to 3% per year [1]. The major clinical manifestations are the outcome of tumor expansion and survival within the bone marrow and resistance to chemotherapy as the final sequel. Basically MM displays a complicated karyotype and high level of genomic/chromosomal instability associated with various gene mutations and chromosomal translocations [1]. On the other hand, oncogenomics studies have found only a few differences that distinguish MM from MGUS [2], because both conditions can present either a hyperdiploid karyotype or a non-hyperdiploid karyotype [3] and similar IgH or IgL chromosomal translocations [4], implying that most above genetic changes may not contribute to MM progression. MM pathogenesis can also be largely explained on the basis of interaction of MM cells with bone marrow microenvironment (BMME) components and signaling pathways thereof leading to MM cells growth and survival, angiogenesis, osteolytic lesions and drug resistance (DR). In this respect, a variety of candidates (genes/proteins) have been identified mostly through gene expression profiling (GEP) studies, which include heat shock proteins (HSPs), some miRNAs, c-MAF, NFκB, Notch and Wnts and their relevant signaling pathways. Perhaps the main feature of these pathways which has made them attractive drug targets is that they are mostly active in MM cells in the context of BMME hence contributing to various aspects of MM pathology especially DR. Furthermore, the new concept of MM cancer stem cell (MMCSC) indicates involvement of Notch and Wnt signaling pathways in biology of this “MM-initiating” cell which takes advantage of bone marrow stromal cells (BMSCs) protection, and indeed new efforts on targeting MMCSC are ongoing [5].

### Drug resistance in malignancies: general concepts

Chemotherapy refractoriness tends to be a clinical frustration in blood cancers and a variety of solid tumors including breast, ovarian, lung, and lower gastrointestinal tract cancers [6-8]. During the past decades, multitudes of mechanisms have been suggested for this DR. For example, drug may be prevented from entering the cells or be pumped out of the cells; it may become enzymatically inactive, mutation or alteration in expression of the drug target, and derangement in mechanisms of apoptosis, senescence and DNA repair could also contribute to DR. Likewise, in hematologic malignancies DR eventually happens although most acute leukemias respond efficiently to chemotherapy at the beginning [8]. Basically, malignant tissues consist of a heterogeneous population of cells with different levels of sensitivity to chemotherapy [7, 9, 10]. Some of these cells may be easily eliminated by the drug while others may become totally resistant. This is in clear contrast to normal cells which usually respond homogeneously to the chemotherapeutic drug. In most cases, malignant cells may become resistant to a single drug in which case DR can be circumvented by using a combination of structurally and functionally different chemotherapeutic drugs. However, cancer cells may also become cross-resistant to various drugs leading to a situation known as multiple drug resistance (MDR). The main mechanism controlling MDR is overexpression of an ATP-dependent efflux pump known as P-gp [11]. This 170KD protein is encoded in humans by *MDR1* gene and is the first known member of ABC (ATP-binding cassette) transporter superfamily. In MDR phenotypes, P-gp is overexpressed and through pumping the drug out of the cells reduces the intracellular concentration of the drug below minimum threshold for effective response, hence rendering cancer cells drug resistant. Other members of transporter superfamily include multi-drug resistance protein-1 (MRP-1), lung resistance related protein (LRP) and breast cancer resistance protein (BCRP). P-gp, MRP-1 and LRP have been found upregulated and associated with DR in acute lymphoid and myeloid leukemia patients leading to those markers being used as targets for MDR modulation [12]. In MDR, it appears that mechanisms controlling drug accumulation inside the cells are defective, most possibly through altering membrane lipids (ceramides) which in turn limit drug uptake or increase drug efflux [13]. Interestingly, sphingosine-1-phosphate (S1P), a metabolite of ceramide, can confer resistance to drugs in hematologic cancers [14, 15]. The outcome of above transformations is inhibiting apoptosis (which is normal mechanism of most anti-cancer drugs), limiting normal processes of drug detoxification and DNA repair, and alteration in mechanisms of cell cycle control and check points. It should be noted that gene mutations of drug transporters or drug receptors could also contribute to MDR phenotype [11, 16].

Notably, the concept of cancer stem cell (CSC) in both solid and hematologic cancers indicates that “cancer initiating cells” resist chemotherapy due to their ability to self-renew, differentiate and remain relatively quiescent, features in fact hampering the effects of chemotherapeutic cytotoxic drugs which typically target rapidly dividing cells [10, 17].

Genetic alterations in signaling pathways downstream to target activation will also have effect on drug response. In most cases, signals will impinge on mutated oncogenes in latter pathways leading to upregulation of survival and drug resistance or downregulation of cell death responses. For example, resistance to Trastuzumab (in treatment of HER-2 positive breast cancer) can be due to upregulation of signaling pathways downstream to HER-2, as a result of *PTEN* loss, or mutations in *PI3K* or *AKT1* [10]. Genomics technology has now deciphered the impact of somatic mutations on some critical oncoproteins including *RAS, EGFR, BCR-ABL* and many others. These somatic alterations cause the tumors rely unusually on a specific molecular pathway or signaling system. This has been referred to as “oncogene addiction”, which is in fact excessive tumor dependence on at least one gain-of-function gene mutation for survival [9].

### Drug resistance in multiple myeloma

In spite of current efficient therapeutic regimens for MM patients, DR is perhaps still the major concern. For instance, bortezomib which continues to be used as a first-in-class drug in MM; many patients may be intrinsically resistant to it or develop resistance in the course of treatment. Although real mechanisms of resistance to bortezomib in MM patients are not yet deciphered, mutation in β5-subunit of proteasome (PSMB5) (conflicting reports), derangement of stress response, survival and antiapoptotic pathways have been indicated to be involved [18]. During the past years many studies were focused on the mechanisms underlying DR in MM, and considering the complex and heterogeneous nature of MM the number of these studies is noticeably increasing. However, with that bulk of research in the past and present, we still don't know exactly how MM progresses from its precursor state, how drug resistant MM clones persist in the presence of effective therapies, and why some MM patients relapse. Here to make a more mechanistic picture of DR in MM based on the most recent findings, we will pursue the discussion covering four categories of explanations: a)-Impact of cytogenetic and epigenetic alterations, b)-Role of deregulated signaling pathways, c)-Role of bone marrow microenvironment, d)-MM cancer stem cell.

### Impact of cytogenetic and epigenetic alterations

MM is universally recognized as having a high level of genomic instability and a very complex cytogenetic constitution which is displayed as changes in both number and structure of various chromosomes [19, 20]. Notably, aberrant homologous recombination (HR) has been identified as the main mechanism in MM genome instability which increases overtime contributing to MM aggressiveness and DR [20]. Based on karyotype changes MM patients are placed in two groups: hyperdiploid (HRD) which have 48-75 chromosomes (involving mostly odd-numbered chromosomes 3,5,7,9,11,15,19,21) and non-hyperdiploid (NHRD) which have less than 45 or more than 75 chromosomes and display IgH translocations or chromosomal deletions such as del (13) [21]. It has been suggested that only 10% of HRD group show a primary IgH translocation at 14q32 locus while it amounts to 70% in NHRD group [21], this might partly explain the better survival and prognosis of HRD group relative to NHRD group. The IgH translocation usually involves juxtaposition of immunoglobulin gene to an oncogene on partner chromosomes creating several reciprocal translocations, including two more frequent ones t(4;14)(p16;q32) in 15% and t(11;14)(q13;q32) in 17% of MM patients [22]. Translocation t(4;14) deranges at p16 locus the expression of FGFR3 which is a proto-oncogenic receptor tyrosine kinase, and multiple myeloma SET domain (MMSET) which apparently has methyl transferase function [23](readers are also referred to ref. [24] for FGFR3 biologic function). Translocation t(11;14) causes upregulation of cyclin D genes which play key role in cell cycle. Of all chromosomal translocations in MM, t(4:14)(p16;q32) has frequently been associated with adverse outcome (and possibly resistance to alkylating agents) in patients under high dose therapy (HDT) modalities or ASCT, but t(11;14) usually confers a favorable prognosis [25-29]. Of important note, several studies confirm that including bortezomib in the treatment regimen of patients with t(4:14) translocation improves and even overcomes the risk factor [30-33]. Less frequent translocations, t(14;16) and t(14;20), which upregulate expression of oncogenes c-MAF and MAFB, respectively, have also been associated with MM adverse clinical outcomes [22]. The deletion 17p13 is one of the most important prognostic markers in MM which is observed in 10% of newly diagnosed patients but increases significantly at later stages [21, 27, 30, 34]. This abnormality is associated with aggressive disease and failed outcome even in the face of novel bortezomib or IMiD-based modalities. In (del) 17p, *TP53* heterozygosity is lost leaving it in a monoallelic form. This mechanism in fact limits the key role of p53 in controlling cell cycle and apoptosis and might partly explain the failed treatment. Additional genetic aberrations include c-MYC rearrangements which occur in 15% of newly diagnosed MM, 40% of advanced MM tumors and almost 90% of human myeloma cell lines (HMCLs), indicating that c-MYC overexpression is a marker of MM progression [35-37]. Compared to MM, c-MYC is almost not detected in MGUS [37, 38], and it has also been implicated in DR in MM [39].

MM patients who relapse or become refractory (primary or after a salvage therapy) carrying any “bad” prognostic cytogenetic marker, might refer with some level of (acquired/intrinsic) drug resistance [40, 41]. While the real impact of cytogenetic aberrations on DR or MM relapse is not mechanistically understood, in some cases experimental studies have yielded helpful clues. For example, in patients with t(14;16) and t(14;20) translocations where MAF genes are overexpressed, it was shown that blocking MEK pathway could downregulate MAF, inhibit cell proliferation and sensitize MM cells to the drugs, indicating that MAF exploits a common pathway in both translocations and MEK would be an additional drug target for above patients [42, 43]. Furthermore, +1q is found in 39% of newly diagnosed MM and in almost 70% of MM patients harboring t(14;16) or t(4:14) translocations, and has been associated with adverse outcome even in intensively treated patients [33, 44, 45]. Studies have shown that the amplified region in chromosome 1q carries the oncogenes *PDZK1* [46] and *CKS1B* [47]. PDZK1 was suggested to be involved in MM cells resistance to several drugs, as its silencing led to increased drug sensitivity. Surprisingly, PDZK1 was first identified through its interaction with several proteins including MRP2 to make a functional cluster involved in MDR phenotype in cancer cells [48, 49]. CKS1B has also been shown to promote MM cells growth and proliferation [50], and to confer DR through MEK/ERK and JAK/STAT3 signaling pathways [51]. On the other hand, 1q21 deletion was also found a risk factor independent of CKS1A amplification in MM patients [52]. Moreover, a group of researchers showed that t(4:14) and t(11;14) translocations, which confer different clinical outcomes, contribute to pathologic complications through different molecular mechanisms and that they share only few mutated genes [53]. Finally, FGFR3 which is overexpressed in t(4:14) translocation was demonstrated to confer resistance to dexamethasone in MM cells [54], and FGFR3 has been used as a drug target in t(4:14)-positive MM cells [25, 55]. However, another group reported that t(4:14) is by itself a risk factor conferring poor drug response independent of FGFR3 expression [25]. Hence, the extent to which above findings can explain DR in relapsed / refractory MM patients with above cytogenetic aberrations is not clear, with most clinical studies suggesting associations (not causes and effects). Nevertheless, gene expression profiling (GEP) and whole genome sequencing (WES) technologies have made possible the establishment of first portraits of gene mutation spectrum and molecular categorization or risk stratification of MM [23, 56-61]. One major goal of these approaches is also finding any collaborative effect of cytogenetic changes and gene mutations on adverse clinical outcome, relapse or DR in MM. *TP53* gene mutations are rare in MM and basically occur at later stages of the disease with strong association with therapy resistance [62-65]. Loss of *TP53* locus in MM has been consistently associated with poor survival and resistance to therapy, and with deregulation of various p53 target genes [34, 66].

Recent investigations have unraveled substantial contribution of epigenetic changes to hematologic cancers progression and pathogenesis (reviews at [67, 68]). Epigenetic changes in human genome occur in two common forms, DNA methylation and chromatin (histon) modifications (acetylation and deacetylation). But much to our surprise, recognition of abnormal DNA methylation in human cancers has a long history [69]. Basically, DNA methylations occur at CpG islands of promoters influencing the expression of various genes which in most cases are oncogenes controlling proliferation, apoptosis, DNA repair and drug sensitivity of malignant cells [70]. In spite of extensive investigations on genetic and cytogenetic alterations in MM, our knowledge of the mechanistic role (s) that epigenetic changes might play in MM drug response is limited. Nonetheless, the role of epigenetic markers in MM pathogenesis and MGUS transition to overt MM is increasingly evidenced, with frequency of some hypermethlyated genes being low at early stages but increasing with MM progression. Using methylation-specific polymerase chain reaction (MSP) most studies have detected hypermethylation of such genes as *p15*, *p16*, *p73* (cell cycle), *DAPK*, *CASP8* (regulation of apoptosis), *SOCS1* (cytokine signaling), *FHIT1* (tumor suppressor gene), O^6^-methylguanine DNA methyl transferase or *MGMT* (DNA repair), *TGFBR2* (growth factor receptor signaling) and *e-cadherin* (cell adhesion) [71-79]. However, latest investigations on MM epigenome using genome-wide methylation arrays have yielded amazing findings shedding more light on epigenetic changes contribution to MM progression and partly DR [80-82]. In these studies, it is shown that the methylation rate increases in transition from MGUS to MM or from MM to plasma cell leukemia (PCL), and that the global methylation pattern in normal B cells, normal plasma cells and MGUS is explicitly hypomethylation. More interestingly, they show that tumor suppressor genes involved in drug response (*TGFB1*) and interaction with bone marrow microenvironment (*SPARC*) are hypermethylated and associated with a short OS [81]. In another recent study, researchers tried to find the association between epigenetic changes and pattern of response to bortezomib in relapsed MM patients. Using DNA methylation PCR, they analyzed CpG island-related DNA methylation profile of 30 genes in 75 relapsed MM patients under bortezomib treatment. They detected a low global methylation status in all patient samples and found that patients with a higher global DNA methylation (more than 3.95% of total DNA methylated) had higher overall survival (OS) than patients with more unmethylated DNA following bortezomib treatment. Furthermore, in gene-specific methylation they found that patients with lower frequency of methylated *NFKB1* and *CXCR4* genes had higher OS and progression free survival (PFS), respectively. Also, lower global DNA methylation and higher *NFKB* methylation pattern conferred a very short OS after bortezomib treatment. Moreover, it has been reported that hypermethylation in *CDKN2A*, *CDKN2B*, *TNF* and *RB* genes is detected more frequently in relapsed MM patients than newly diagnosed MM patients [83]. Although, in above studies little focus is on the functional and mechanistic aspect of epigenetic changes to MM biology especially in terms of drug response, they convincingly recognize some epigenetic markers as promising drug targets. On the other hand, an association of DNA methylation in MM with drug (dexamethasone) resistance has only been reported in one study [84]. They showed that hypermethylation of *RASD1* gene in MM cells was associated with resistance against dexamethasone and treating the cells with 5'-aza-cytidine sensitized the cells to the drug. Additionally, most of above gene methylations have also been reported in acute lymphoid and myeloid leukemias, and methylations of HIC1 and WIT1 were associated with late stage AML and chemotherapy resistant AML, respectively [12]. Indeed some epigenetic markers are being considered as interesting drug targets in hematologic malignancies [85]. It should be noted that histone modifications also have known roles in cancer, but we still don't have sufficient evidence to support role of chromatin changes in MM pathogenesis. However, histone deacetylase inhibitors (HDACi) alone or in combination with chemotherapeutic drugs have always shown profound anti-myeloma activities in both *in vitro* and *in vivo* assessments [85-89]. Taken together, how epigenetic changes in MM might affect drug response of the patients and whether they play role in DR of MM is not quite understood. One general inference from all above studies could be, at least partially, involvement by epigenetic changes of some pathways controlling cell cycle and proliferation. This idea is rather supported by the study at ref. [90]

### Role of deregulated signaling pathways

To elucidate the biological and biochemical mechanisms underlying DR of MM cells, various researches have been focused on signaling molecules, scaffolds, complexes and mediators in a variety of signaling pathways to reveal targets which could possibly mediate DR following drug treatment. These signaling pathways are mostly involved in two main DR-associated mechanisms: aberrant drug transport, anti-apoptosis.

### aberrant drug transport: MDR phenotype

Overexpression of P-gp, the product of *MDR1* gene, has frequently been observed in MM and strongly associated with relapse and DR [91-94]. Indeed, several MDR modulators have already been applied to MM clinical trials but were mostly associated with poor benefit due to high toxicity [95-97]. Apparently, polymorphisms in *MDR1* gene (SNPs) will also influence therapy outcome, for instance in MM patients under DAV protocol [98], and variously detected in MGUS, MM and relapsed MM tumor cells [99], however, their contribution to bortezomib resistance is not clear [98, 100]. In contrast, a recent study confirms that bortezomib functions as a substrate for P-gp and overexpression of P-gp could underlie bortezomib resistance [101], but this finding is apparently not supported by other studies [18]. Surprisingly, another recent study shows that overexpression of P-gp (ABCB1) defines a subpopulation of MM cells which are resistant to carflizomib, the newly FDA-approved second generation proteasome inhibitor [102]. The major concern with MDR markers is that they are weakly expressed at diagnosis but overexpressed after chemotherapy, for instance, almost 6% of newly diagnosed MM patients but more than 43% after chemotherapy (vincristine and doxorubicin) were P-gp positive [94]. Moreover, it has been suggested that following treatment with MDR modulators, tumor cells could upregulate drug target proteins or create mutations abrogating drug-target interaction [97]. The other MDR-related protein, breast cancer resistance protein (BCRP, ABCG2), seems not to play role in MDR in MM [103], but *ABCG2* gene has been found methylatd and upregulated following chemotherapy [104]. On the other hand, a new study on MM cell biology and pathogenesis identified a chromosomal instability (CIN) gene called *NEK2* whose overexpression upregulated MDR-related proteins, MDR1 (P-gp), MRP1 and BCRP and was strongly associated with resistance to drugs (bortezomib and doxorubicin), rapid relapse and poor outcome in MM [105].

### anti-apoptosis mechanisms

*p53 tumor suppressor protein*, known as” guardian of genome”, performs an outstanding task in controlling cell cycle, apoptosis, and DNA repair, senescence and autophagy [106-109]. p53 is inactivated in a variety of human cancers, including in 10-12% of MM tumors mostly due to loss of heterozygocity [110, 111]. In many cancers, however, p53 inactivation could also be the result of mutations in p53-DNA binding domain or through overexpression of murine double minute 2 (MDM2) [108]. MDM2 is an E3-ubiquitin ligase which binds p53 to ubiquitinate and target it for degradation through ubiquitin/proteasome pathway [112], and is overexpressed in 58% of MM samples which has been frequently associated with chemoresistance [113, 114]. Of important note, MDM2 in many cancers remains an oncogene even in the face of functional p53, and overexpression of MDM2 in cell lines culminated in resistance to vincristine, doxorubicin and etoposide (e.g. see the ref. [115]). It has also been suggested that MDM2 imposes DR effects through increase in p53 degradation or interaction with *MDR1* gene [113]. Another tumor suppressor p14ARF binds to MDM2 and sequesters it in the nucleus to allow p53 stabilization [116]. The regulatory loop p14ARF-MDM2-p53 plays substantial role in cell fate in cases of cellular stresses such as DNA damage (e.g. following chemotherapy) [116]. As happens in MM cells [114], deletion/mutation in p53 or p14ARF (loss of function) or upregulation of MDM2 would direct the pathway toward deregulation of p53-related signaling pathways and downstream targets (p21, GADD45, Bax, Noxa, Puma), hence development of anti-apoptotic and DR signals. Targeting p53 in MM has been considered as an interesting treatment strategy based on mostly restoring p53 function in MM cell lines and primary cells harboring mutated p53. As a nongenotoxic approach, disruption of p53-MDM2 interaction using nutlin-3 or RITA triggered apoptosis in MM cells where synergistic effects with bortezomib were also observed [117-121]. These studies provided evidence that p53 protein and its pro-apoptotic targets Bax, Puma and Bak were upregulated but anti-apoptotic Bcl-2 was downregulated following above therapeutic modulation. However, latter findings were not evidenced in primary tumor cells from relapse or refractory MM patients. A recent study demonstrated that inhibition of ubiquitin-specific protease-7 (USP7), which normally stabilizes MDM2, triggers apoptosis in bortezomib-resistant MM cells, confirming the idea of p53 downregulation as a DR mechanism in MM [122]. Involvement of p53 in apoptosis induced by nucleoside analogs (gemicitabin and clofarabin) in MM cells was also shown by another recent study [123]. Several proteins have been described with their functions to be p53-dependent, including RPRM (reprimo), a newly identified candidate mediating cell cycle arrest by p53 [124]. Interestingly, *reprimo* gene has been reported to be methylated in MM [125]. Two members of p53 family, p63 and p73, bear high level of homology to p53 but their contribution to MM pathogenesis, progression and therapy outcome has not been fully investigated, however, p73 mutations and hypermethylations have been reported in MM cells especially at advanced stages [74]. Furthermore, one study demonstrated accumulation of p63, p73 and p53 in the nuclei of MM cells following drug-induced DNA breaks [126]. Early growth response-1 (EGR1) gene which functions in a p53-dependent manner [127, 128], was shown to mediate *JUN-* induced apoptosis in MM cells and be associated with poor outcome and DR when downregulated [129]. All above studies highlight the key role of p53 and its related proteins in MM pathogenesis and drug response.

*The transcription factor NFκB* is a well-known player in MM pathogenesis and biology in terms of tumor cell proliferation, expansion and DR [1, 130, 131], and targeting NFκB pathway in MM (e.g. using bortezomib) has recently improved MM therapy [1, 132]. The contribution of NFκB pathway to MM pathogenesis is its constitutive activation in a large proportion of MM tumor cells and HMCLs mostly due to ligand-dependent activation, including effect of TNF-α, TRAIL, BAFF and CD40L on MM cells inside the bone marrow [1]. The latter cytokines are secreted by BMSCs and play critical roles in MM clones maintenance and DR. However, it has recently been discovered that mutations in some genes of NFκB platform result in constitutive activation of NFκB pathway making MM cells less dependent on BMME protection and more refractory to chemotherapy [56, 133, 134]. These findings create novel insights into role of NFκB pathway activation in MM pathogenesis but cannot underscore the contribution of extrinsic signals in BMME to NFκB activation-related MM complications especially DR [135](and see discussion below). Intriguingly, it is also specified that MM cells harboring *TRAF3* gene mutation in NFκB pathway are resistant to dexamethasone but sensitive to bortezomib [133]. This is in line with fact that apoptotic function of bortezomib is partly explained by blocking canonical NFκB pathway; however, it unexpectedly induces the alternative (non-canonical) pathway making MM cells less responsive [136]. Moreover, in some cases MM cells may develop a bortezomib-resistant NFκB phenotype through a proteasome-inhibitor resistant (PIR) pathway [137]. The latter flaws of bortezomib may explain to a large extent why it should be applied in combined regimens for those MM patients who are in relapse or refractory to it. The transcription factor NFκB could also contribute to DR in MM through upregulation of some BCL-2 anti-apoptotic family members including BCL-XL [138]. A variety of other molecules and signaling pathways have been implicated in anti-apoptosis or DR mechanisms of MM cells (table 1).

**Table 1:** Other molecules/pathways with demonstrated roles in MMDR

Samples analyzed	Molecule/pathway	Alteration pattern	Functional outcome	Refs.
HMCLs, primary cells, in vivo mouse models	HSPs (HSP90, HSP70, HSP72, HSP-27, HSF-1) HSPs function as buffering systems to safe guard various client targets (e.g. oncogenes) and signaling pathways (e.g. apoptosis) involved in cell survival.	Overexpression, especially following adhesion of MM cells to BMSCs, and possibly through the effect of IL-6.	Increase in growth, proliferation, DR, CAM-DR and resistance to apoptosis mainly through activated STAT3, NFκB, Akt and MAPK pathways. Some HSPs (HSP27, HSP70) are upregulated following treatment of MM cells with proteasome inhibitors due to induction of stress response inducing DR and HSP90 inhibitors show synergism with bortezomib. In MM, HSPs can also stabilize antiapoptotic BCL-2 members (BCL-2, MCL-1 and BCL-XL), as HSP-90 inhibition in U266 cells resulted in significant apoptosis and downregulation of above proteins.	[139-147]
HMCLs, primary cells, in vivo mouse models	Notch signaling pathway	Overexpression of Notch receptors (Notch1) on MM cells and of Notch ligands (Jagged-1, Dll1) on BMSCs. Constitutive activation of the pathway in BMME.	The Notch1-jagg1 pathway is activated due to MM cell-BMSC adhesion inducing CAM-DR. Inhibition of Notch signaling by GSI (Γ-secretase inhibitor) induces MM cells apoptosis through upregulation of Noxa. Dll1 /Notch pathway also promotes MM cells resistance to bortezomib through upregulation of of CYP1A1, a Cytochrome P450 enzyme. Latter pathway is also constitutively activated in MMCSCs (CD138- cells) which show more resistance to drugs than CD138+ cells through upregulation of BCL-2, MCL-1 and BCL-XL. Notch pathway may contribute to MMDR through MM cells-osteoclast interaction.	[148-154]
HMCLs, primary cells,	Wnt signaling pathway	Overexpression of Wnt receptors on MM cells and, like Notch pathway, constitutive activation of the pathway within BMME, partly due to hypermethylation of some Wnt antagonists.	Activation of Wnt/β-catenin (canonical) pathway in MM cells induces tumor growth, proliferation and metastatic features, mediates CAM-DR of MM cells to lenalidomide or doxorubicin.	[155-160]
HMCLs, clinical studies	Cereblon (CRBN)	Downregulation due to gene mutation.	First identified as the primary target of teratogenicity in thalidomide. Associated with resistance to IMiDs (lenalidomide), high expression of CRBN is a favourable marker in MM patients under IMiDs protocol. In a case study of advanced (extra medullary) MM with a MDR phenotype, CRBN was found to be mutated.	[161-166]
HMCLs, MM primary cells, in vivo mouse models	Telomerase	Hyperactivity of telomerase, partly due to co-operation of KRAS and RB1 oncogenes with telomerase main gene hTERT.	Maintenance of telomere length leading to MM cell proliferation, survival and drug resistance. Hyperactivity of telomerase has been reported in a large number of relapsed, refractory or newly diagnosed MM patients, and was suggested to indicate a poor prognosis. A possible mediator of bortezomib resistance.	[167-171]
HMCLs, MM primary cells, in vivo mouse models, clinical studies	miRNAs	Aberrant expression, up-or-downregulation, possibly by epigenetic mechanisms.	Correlation with patient survival. Down-or-up-regulation of several miRNA (e.g. miRNA-21) in drug resistant HMCLs compared with drug sensitive parent lines. Induction of BMME-related DR by upregulated miRNAs, miRNA-21,-19a and 19b, or by downregulated miRNAs, miRNA-15/16a (possibly through IL-6 upregulation or SOCS1 downregulation). Overexpression of some oncogenes including CCDN1, TACC3, MAFB, FGFR3 and MYC by other downregulated miRNAs (miRNA-425, miRNA-152, miRNA-24). Inactivation of p53 protein or its related targets by downregulated (miRNA-214) or upregulated (miRNA-125b/25b/30d and miRNA-181a,b/32) miRNAs.	[172-183]
HMCLs, MM primary cells	Krüppel-like factor 4 (KLF4)	Overexpression in MM patients harboring t(4;14)(p16.3;q32), not expressed in HMCLs due to DNA methylation.	High expression of KLF4 was associated with upregulation of p27Cip1 abd p27Kip1 and conferred resistance to melphalan but not bortezomib.	[184]
HMCLs, MM primary cells	S1P (Sphingoside-1-phosphate)	Overexpression	Possibly upregulated by IL-6, S1P confers antiapoptosis and DR signals through MCL-1 upregulation.	[14, 15, 185, 186]
HMCLs, MM primary cells, in vivo mouse models, clinical studies	NEK2	Overexpression	Highly correlated with rapid relapse, DR and poor outcome. Induction of DR mainly through interaction with drug efflux pumps.	[105]
HMCLs, patient primary cells	NRAS, KRAS, BRAF	Gene mutation	Induce DR in MM cells by triggering MAPK/ERK pathway	[56, 187-189]

### Role of bone marrow microenvironment

The role of BMME components especially BMSCs and extracellular matrix (ECM) protein in the pathogenesis of MM has been the focus of a good deal of research and to our best knowledge contribution of BMME is noteworthy (reviews at [1, 190, 191]. The fundamental element in this performance is a well-established adhesion between MM cells and BMSCs or fibronectin leading to maintaining the growth, proliferation, invasion and DR of MM clones and also promoting bone lesions and angiogenesis. The best explained mechanisms of MM drug/apoptosis resistance due to BMME effects in MM are soluble factor-mediated drug resistance (SFM-DR) and cell-adhesion mediated drug resistance (CAM-DR) which are in fact two forms of environment-mediated drug resistance (EMDR) in cancers [192]. SFM-DR can be better explained by involvement of IL-6, the most critical growth and survival factor for MM cells, and CAM-DR is mediated by adhesion of MM cells to BMSCs or ECM proteins involving adhesion molecules (β1 integrins). However, other cytokines including HGF [193] and IGF-1 [194] have also been implicated in MMDR. It was shown that bortezomib-resistant myeloma cell lines and clinical samples from bortezomib-refractory MM patients displayed an activated IGF-1/IGF-1R signaling pathway and a high level of IGF-1 cytokine which were associated with bortezomib resistance. Blocking IGF-1R or IGF-1 signaling alone or synergistically with bortezomib increased MM cells death. Intrinsic (*de novo*) resistance to drugs including bortezomib may occur in some MM patients, which has been suggested to arise largely due to CAM-DR (a transient form of DR only during cell-cell or cell-ECM adhesion) and more importantly may contribute to emergence of *acquired* DR in the course of treatment [195]. The same concept has also been supported as an underlying mechanism for *de novo* DR and minimal residual disease in various cancers [192, 196]. Following adhesion of MM cells to FN or BMSC, IL-6 is secreted by BMSCs or by MM cells in an autocrine manner [197-199]. IL-6 is certainly the best studied cytokine critical to MM cells, which has been implicated in MM cells resistance to various apoptotic signals including Fas/Apo-1 and chemotherapeutic drugs [200-202], with these responses being possibly controlled through Jak/STAT signaling pathway as was shown in U266 cell line [203]. It has also been demonstrated in MM primary samples that MM cell clones with autocrine IL-6 signal are more resistant to dexamethasone than those with no autocrine IL-6 signal [204]. Additionally, blocking IL-6 receptors with CNTO 328 increased sensitivity of MM cells to bortezomib [205]. These observations highlight the crucial role of IL-6 in anti-apoptosis and DR mechanisms in MM.

CAM-DR to doxorubicin, melphalan, vincristine, bortezomib and mitoxantrone has been induced in MM cell lines and patient primary cells through adhesion to FN or BMSCs which was mostly mediated by VLA4 integrin (α4β1) [206-209] and also through LFA-1 [210]. It should be noted that other integrin molecules including β7 and VLA5 (α5β1) could also play role in CAM-DR in MM cells [207, 211]. More importantly, MM primary cells with a higher expression of adhesion molecules (VLA4 and ICAM-1) are drug resistant and believed to be selected by the chemotherapy during treatment tipping contribution to *acquired* DR by CAM-DR [212]. Of note, *acquired* DR is genetically much more complex than *de novo* DR (CAM-DR) and indeed takes a long time to emerge. To examine this idea, Hazlehurst et al. induced *de novo* DR to melphalan in RPMI8226 cell line through adhesion to FN and also in patient primary cells to confirm the clinical relevance [213]. They then established an acquired melphalan-resistant RPMI8226 subline (RPMI8226-LR5) through long-time drug exposure. Using oligonucleotide microarray, they detected change in the expression of 1479 genes in *acquired* DR model compared with only 69 genes in *de novo* DR parent line. These findings strongly indicate that unlike *acquired* DR which is associated with an outstanding transcriptome change, *de novo* DR (CAM-DR) is mostly regulated by post transcriptional mechanisms. Indeed, induction of β1-integrin mediated CAM-DR in MM cells was characterized by G1 cell cycle arrest accompanied by an increase in p27^Kip1^ protein level and decrease in enzymatic activity of cyclin A and cyclin E [208], upregulation of p21^Cip1/Waf1^ [149] or downregulation of Bim (the apoptotic BCL-2 family member) [213] implying involvement of posttranscriptional mechanisms. Furthermore, adhesion through β1 integrin also induces resistance to apoptotic signals such as Fas/Apo-1 in various hematopoietic cancer cell lines including MM with a post-transcriptionally regulated mechanism. Observations in one study indicated that CAM-DR in MM cells was associated with increase in solubility and redistribution of c-FLIP_L_ allowing its binding to and inhibiting death-inducing signaling complex (DISC) (which forms following CD95 ligation) and thus blocking apoptosis [214].

**Figure 1:**
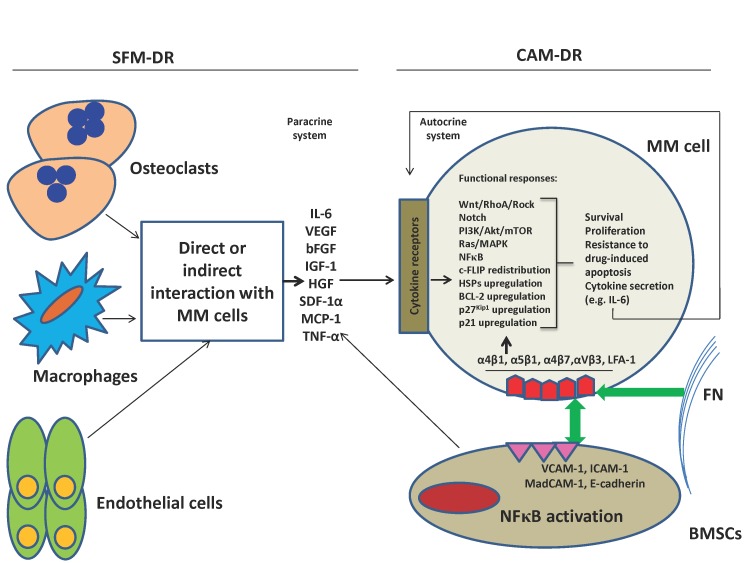
SFM-DR and CAM-DR work usually together within the bone marrow environment Adhesion of MM cells to BMSCs and FN through integrin molecules triggers a variety of signaling pathways (including Ras/MAPK, PI3K/Akt, NFκB, Notch, Wnt, HSPs) involved in cell proliferation, anti-apoptosis, DR and cytokine secretion (IL-6), and also upregulation of anti-apoptotic BCL-2 family members BCL-2, MCL-1 or BCL-XL. The above adhesion induces secretion of several cytokines (IL-6, VEGF, HGF, IGF-1, SDF-1α, TNF-α, MCP-1) by BMSCs leading to triggering most of above pathways and induction of resistance to apoptosis and drugs in MM cells (paracrine). IL-6 can also stimulate MM cells in an autocrine manner, although this system has been shown for some other cytokines as well. Furthermore, most above cytokines may also be secreted by osteoclasts, endothelial cells and macrophages during their direct or indirect interactions with MM cells leading to triggering of above functional responses in latter cells.

As a matter of fact *in vitro* analysis of CAM-DR and SFM-DR as two separate systems is oversimplification, because it is not unexpected to think that two processes work hand in hand inside the bone marrow (figure 1). In line with this, one study demonstrated the synergistic anti-apoptosis resistance effect of adhesion to FN (β1 integrin signaling) and IL-6 (gp130 signaling) in MM cells associated with activated STAT3 signaling pathway [199]. Moreover, treating MM cells with HGF increased their adhesion to FN which was mediated by VLA4 integrin and PI3K and NFκB pathways implying a synergistic effect of FN-adhesion and HGF in promoting CAM-DR of MM cells [193]. More interestingly, it is suggested that SFM-DR and CAM-DR confer resistance to drug-induced apoptosis in MM cells through distinct mechanisms [215].

However, in recent years many other studies have been focused on deciphering molecular mechanisms of drug/apoptosis resistance in MM conferred by bone marrow stroma. Adhesion of MM cells to BMSCs induces upegulation of Notch receptors on former cells which bind to their specific ligands (Jagged) on latter cells culminating in resistance to drug-induced apoptosis [149]. BMSCs were shown to induce bortezomib-resistant NFκB activity in MM cells which was mediated by soluble factors including IL-8 from BMSCs [216], providing a supportive explanation to bortezomib resistance in a large number of MM patients [137]. HSPs have also been implicated in CAM-DR of MM, as adhesion of MM cells to BMSCs and FN upregulated HSP-70 in MM cells, and blocking HSP-70 resulted in increase in melphalan-induced apoptosis and reversed CAM-DR [144]. Co-culture of HMCLs (U266 and NCI-H929) with MM patient BMSCs upregulated survivin in MM cells conferring resistance against daunorubicin [217]. Notably, blocking survivin had already been associated with growth inhibition of MM cells and decrease in their resistance to doxorubicin, melphalan and dexamethasone [218]. CD44 the receptor for hyaluronic acid (HA) mediated resistance to lenalidomide in MM cells and lenalidomide-resistant MM cells adhered more strongly to BMSCs and HA implying a role for CD44 in CAM-DR of MM cells [219]. Upregulation of B7-H1 molecule on MM cells following their adhesion to BMSCs was associated with resistance to dexamethasone and melphalan in MM cells and increase in Bcl-2 and FasL levels [220]. Of note, B7-H1 is also considered as an immune response evasion tool expressed on malignant plasma cells [221].

Some recent studies have also tried to understand if chromosomal translocations and gene mutations might control interaction of MM cells with BMME components and hence trigger CAM-DR. Knock-down of the MMSET protein in MM cells harboring t(4;14) led to decrease in MM cell proliferation and induction of apoptosis (activation of caspase-3 and caspase-9) and changed two genes (*DSG2* and ADAM9) involved in cell-cell adhesion [222]. Although drug sensitivity of MMSET-silenced MM cells was not examined in this study, their findings imply a possible involvement of MMSET in controlling CAM-DR of MM. Using whole exon sequencing (WES), another study detected in MM patient samples and HMCLs somatic mutations in adhesion molecules involved in interaction with BMSCs giving an implication for role of these mutations in CAM-DR, however; no functional experiment was performed [223]. Mutations in some oncogenes including *RAS* may also affect interaction of MM cells with BMME and hence induce CAM-DR [188]. Furthermore, overexpression of c-MAF was detected in 50% of MM primary samples and also in HMCLs lacking c-MAF translocations and was associated with increase in *ITGB7* (integrin β7) gene which is involved in adhesion of MM cells to BMSCs and induction of CAMDR [211, 224].

It is interesting to remember that interaction of MM cells with other cells in BMME could also determine chemoresistance of in MM (figure 1). Osteoclasts confer resistance to doxorubicin-induced apoptosis in MM cells through secretion of IL-6 and OPN (ostepontin) form osteoclasts. These protective effects were largely dependent on direct cell-cell contact [225]. Contribution to chemoresistance has also been implicated by interaction of MM cells with bone marrow endothelial cells, mainly through induction of cytokines such as IL-6, SDF-1α, MCP-1, Ang-1, bFGF and TNF-α by endothelial cells [226]. Macrophages have been recently implicated in chemoresistance in the context of MM bone marrow environment. These cells also confer resistance to apoptosis induced by dexamethasone and melphalan through direct cell-cell contact and by hampering apoptotic caspase pathway [227] and through adhesion molecules PSGL1/selectins and ICAM-1/CD18 in *in vitro* and *in vivo* (SCID mice) via Src, Erk1/2 kinases and c-myc pathways [228]. Some research groups have put forward the role of myeloma bone marrow hypoxia in disease progression and chemoresistance [229]. The mainstay of this concept is that MM bone marrow is more hypoxic than normal bone marrow, and this leads to upregulation of HIF-1α and HIF-2α which has been associated with suppression of p53 in some cancer cells, however; this association in MM is not clear yet. Finally, the role of infectious / inflammatory environment of MM cells in disease pathogenesis and DR has recently become an interesting research focus. The first clue of latter concept was expression by MM clinical samples and HMCLs of a wide range of functional Toll-like receptors (TLRs) [230-232]. These molecules belong to a family of pattern recognition receptors which control and integrate immune responses and have been shown to play multiple roles in cancer pathogenesis. Interestingly, stimulating MM cells with some TLR ligands induced secretion of IL-6, dexamethasone resistance, growth and proliferation [231, 232], upregulation of immune evasion markers [221], or differentially modulated expression of adhesion molecules (α4, β7 and αVβ3) and adhesion to FN [233]. On the other hand, stimulation of some TLRs could also trigger apoptosis in some MM cells and sensitize them to bortezomib in FN context [233]. Taken all together, the role that BMME plays in pathogenesis and chemoresistance of MM is undoubtedly vital, to the extent that a plethora of experimental findings pinpoint diverse cellular and molecular mechanisms involved in this BMME-induced protective shield for MM clones.

### MM cancer stem cell and DR

The bulk malignant cells in MM are plasma cells expressing syndcan-1 (CD138), but this marker is pertinent to terminally differentiated cells with limited proliferative potential, and this for many years has led researchers to explore real “MM-initiating cells” or MM cancer stem cells (MMCSCs). Malignant plasma cells in MM harbor somatically hypermutated immunoglobulin genes remaining constant throughout the clinical course of the disease and do not show intraclonal diversity implying that MM arises from a post-germinal center B cell having already experienced antigenic challenge in lymph nodes. Generally speaking, the CSC model is based on the concept that cancers constitute a hierarchical organization like the hematopoietic system, suggesting that CSC should maintain cancer cells population through an asymmetric division (each CSC produces a daughter and another CSC).

A convincing line of evidence confirms that MM cells contain a rare subpopulation which is clonotypic and drug resistant, expresses phenotypic markers of memory B cell-like and possess stemness features [5, 234-236]. It has been shown that: a)-only CD138^−^ MM cells have the clonogenic potential and are able to propagate MM tumor in NOD/SCID mice, b)-MMCSCs carry the immunophenotype signature CD138^−^/CD19^+^/CD20^+^/CD27^+^, indicating that MMCSCs possess a memory B cell-like phanotype signature arising from a hierarchical pre-malignant plasma cell stage, c)-In a novel 3D model drug resistant MM cells were CD20^+^ [237], and MMCSCs growth was inhibited by rituximab (anti-CD20 mAb), d)-MMCSCs (CD138^−^ MM cells) are resistant to dexamethasone, lanalidomide, bortezomib, are enriched in a side population (SP) with high ALDH1 activity and drug efflux pump [238], and recently e)-CD138^−^ MM cells are ALDH^+^, have a higher clonogenic potential than CD138^+^ cells and are able to expand tumor in NOG mice [239]. Several previous studies from Pilarski et al. also confirmed presence of clonotypic B cells in MM patients contributing to tumor expansion, relapse and DR [240-242]. On the contrary, another group of researchers claimed that MM initiating cells were enriched in the CD138^−^CD19^−^CD38^++^ component [243]. They showed that CD19^+^ MM cells could not produce tumor colonies and engraft SCID-rab mice, while these happened only with CD138^−^CD19^−^CD38^++^, indicating that CD138^−^ MM cells at least in some MM patients are not B cells. Of striking importance, MMCSCs express also functional markers such as drug efflux pumps (ABCC3), ALDH1 and RARα2 which have been associated with clonogenic potential and resistance to chemotherapy further highlighting their contribution to DR and relapse in MM patients [238, 244, 245]. It was demonstrated that overexpression of RARα2 renders MMCSCs (CD138^−^ MM cells) drug resistant by activating ABCC3 gene through stem cell related pathways Hh (hedgehog) and Wnt. Indeed MMCSCs express mRNA of Hh receptors which are normally involved in Hh signaling and regulate homeostasis and biology of normal stem cells [246]. Interestingly, Hh pathway was found active in MMCSCs and blocking this pathway inhibited clonal expansion and induced differentiation. However, activation of this pathway was suggested to be mostly ligand-dependent with BMSCs being the main source of Hh ligands [247], and it can be speculated that interaction of MMCSCs with BMSCs maintains activation of Hh signaling and tumor dominance. Of note, inhibitors of Hh pathway have already entered clinical trials in MM [248]. Moreover, MMCSCs can also express telomerase which has a prominent role in controlling normal stem cell biology and cancer DR [249]. The inhibitor of telomerase activity Imetelstat blocked MMCSCs clonogenic potential *in vitro* and *in vivo*, triggered differentiation of CD138^−^ cells into CD138^+^ cells and decreased the number of ALDH1^+^ cells. Taken all together, the above studies provide strong evidence for existence of well-defined stem/progenitor cells possessing the three prominent features common to CSCs in all cancers: self-renewal, proliferation and drug resistance.

### MMDR in the face of new therapeutic protocols: can we overcome it?

Most therapeutic approaches to date for relapsed or refractory MM patients have been based on combined formulations. Although emergence of new drugs has revolutionized therapy for MM patients, almost all of them finally develop relapse or DR. As discussed above, using advanced genomics / oncogenomics a variety of factors have been identified to play role in this refractoriness, including various gene mutations, overexpression of MDR genes, epigenetic changes and aberrant activation of various pathways. Importantly, new proteasome inhibitors (including Carfilzomib, ONX 0912, MLN 9708, Marizomib), HDAC inhibitors (e.g. Tipifarnib), new IMiDs (Pomalidomide), kinase inhibitors (especially inhibitors of mTOR and HSP90), new immune-based therapies (antibodies against CS-1, CD38, IL-6) and new alkylators (Bendamustine) have proved effective mostly in combination with conventional drugs in phase I and II clinical trials for MM patients who were bortezomib-resistant or in relapse (for reviews see [40, 250, 251]). However, development of DR in these contexts has also been reported necessitating establishment of highly specific targeted therapies. For instance, HSP90 inhibitors prove to be effective with other drugs (including bortezomib), but may induce upregulation of HSP-70 or HSP-27 leading to DR and requires including the inhibitors of latter HSPs [141, 252]. We now know that some genetic alterations detected by oncogenomics with explained roles in MM pathogenesis are also detected in MGUS indicating their poor contribution to disease progression or possibly DR. On the other hand, our knowledge of the role of BMSCs or ECM proteins in MMDR (in terms of their contribution to *de novo* or *acquired* DR), and of the nature of MMCSCs has noticeably increased. Therefore, it is expected that new drugs with the specific ability to target these two elements of MM physiology be also developed in order to eradicate MM initiating clones or decrease the chance of DR or relapse. Interestingly, a recent thorough investigation identified CD138^−^ MM cell subsets harboring Xbp1s^−^ tumor B cells (plausibly MMCSCs) that were bortezomib resistant and targeting latter population has been suggested to be a promising target [253]. Xbp1s has a critical function in B cell development and commitment to plasma cells and is also an important element of unfolded protein response (UPR) in endoplasmic reticulum (ER) stress-related apoptosis.

## CONCLUDING REMARKS AND FUTURE PROSPECTS

Recent progresses in understanding the molecular biology, molecular categorization and therapy of MM patients are certainly amazing. This achievement has been undoubtedly made through precious contribution of advanced technologies such as gene expression profiling (GEP), whole genome sequencing (WGS) or whole exon sequencing (WES), and a tremendous number of *in vitro* and *in vivo* investigations. However, MM is still dealt with as a hard to treat hematologic malignancy just due to *de novo* or *acquired* DR, indicating that some patients may become initially resistant to the drugs or develop DR in the course of treatment. This will explicitly imply that there still exist complexities in pathogenesis and progression of MM warranting unstoppable research. Nevertheless, identification of a variety of molecules and signaling pathways with different levels of contribution to MM pathogenesis has opened a new horizon to much more targeted therapy than conventional cytotoxic chemotherapy. For the most part, novel insights into role of BMME components especially BMSCs and ECM proteins in MM pathogenesis, progression and DR, and also increased understanding of nature and role of MMCSCs in tumor expansion and survival have provided us with more promising therapeutic venues to target vital pulses of MM through development of new agents.

## ACKNOWLEDGEMENT

The study was supported in part by the grants from Leukemia & Lymphoma Society of Canada, Cancer Research Society, International Collaboration Fund from Chinese Ministry of Science and Technology (NO. 2011DFA32820) and Gan-Po 555 project, Jiangxi, China

### Conflict of Interest

The authors declare no conflict of interest
