# Drug resistance in multiple myeloma: latest findings and new concepts on molecular mechanisms

**DOI:** 10.18632/oncotarget.3810

**Published:** 2013-11-24

**Authors:** Jahangir Abdi, Guoan Chen, Hong Chang

Erratum: Drug resistance in multiple myeloma: latest findings and new concepts on molecular mechanisms

Jahangir Abdi^1,2^, Guoan Chen^3^, Hong Chang^1,2,4^

Oncotarget. 2013; 4:2186-2207

PMID: 24327604

http://www.impactjournals.com/oncotarget/index.php?journal=oncotarget&page=article&op=view&path%5B%5D=1497&path%5B%5D=1760

The affiliation of the first author is incorrect in the published paper.

^1^ Division of Immunopharmacology, Utrecht Institute for Pharmaceutical Sciences, Utrecht University, Utrecht, The Netherlands

The corrected affiliation is provided here.

^1^ Dept. of Laboratory Medicine & Pathobiology, University of Toronto, Ontario, Canada

